# The Scavenger Protein Apoptosis Inhibitor of Macrophages (AIM) Potentiates the Antimicrobial Response against *Mycobacterium tuberculosis* by Enhancing Autophagy

**DOI:** 10.1371/journal.pone.0079670

**Published:** 2013-11-04

**Authors:** Lucía Sanjurjo, Núria Amézaga, Cristina Vilaplana, Neus Cáceres, Elena Marzo, Marta Valeri, Pere-Joan Cardona, Maria-Rosa Sarrias

**Affiliations:** 1 Innate Immunity Group, Fundació Institut d’Investigació en Ciències de la Salut Germans Trias i Pujol (IGTP), Badalona, Spain; 2 Unitat de Tuberculosi Experimental (UTE), Fundació Institut d’Investigació en Ciències de la Salut Germans Trias i Pujol (IGTP), Badalona, Spain; 3 Universitat Autònoma de Barcelona (UAB), Bellaterra, Spain; 4 Centro de Investigación Biomédica en Red en Enfermedades Respiratorias (CIBERES), Instituto Carlos III, Palma de Mallorca, Spain; 5 Microscopy Platform, Vall d’Hebron Research Institute (VHIR), Barcelona, Spain; 6 Centro de Investigación Biomédica en Red en Enfermedades Hepáticas y Digestivas (CIBERehd), Instituto Carlos III, Barcelona, Spain; Bose Institute, India

## Abstract

Apoptosis inhibitor of macrophages (AIM), a scavenger protein secreted by tissue macrophages, is transcriptionally regulated by the nuclear receptor Liver X Receptor (LXR) and Retinoid X Receptor (RXR) heterodimer. Given that LXR exerts a protective immune response against *M. tuberculosis*, here we analyzed whether AIM is involved in this response. In an experimental murine model of tuberculosis, AIM serum levels peaked dramatically early after infection with *M. tuberculosis*, providing an *in vivo* biological link to the disease. We therefore studied the participation of AIM in macrophage response to *M. tuberculosis in vitro*. For this purpose, we used the H37Rv strain to infect THP-1 macrophages transfected to stably express AIM, thereby increasing infected macrophage survival. Furthermore, the expression of this protein enlarged foam cell formation by enhancing intracellular lipid content. Phagocytosis assays with FITC-labeled *M. tuberculosis* bacilli indicated that this protein was not involved in bacterial uptake; however, AIM expression decreased the number of intracellular cfus by up to 70% in bacterial killing assays, suggesting that AIM enhances macrophage mycobactericidal activity. Accordingly, *M. tuberculosis*-infected AIM-expressing cells upregulated the production of reactive oxygen species. Moreover, real-time PCR analysis showed increased mRNA levels of the antimicrobial peptides cathelicidin and defensin 4B. These increases were concomitant with greater cellular concentrations of the autophagy-related molecules Beclin 1 and LC3II, as well as enhanced acidification of mycobacterial phagosomes and LC3 co-localization. In summary, our data support the notion that AIM contributes to key macrophage responses to *M. tuberculosis*.

## Introduction

The causal agent of tuberculosis (TB), *M. tuberculosis* is a human pathogen infecting over a billion people worldwide. The relevance of this disease for human health is reflected by the following figures. In 2011 alone, 8.7 million people fell ill with TB and 1.4 million died from the disease (Global tuberculosis report, 2012, World Health Organization, WHO. Available: http://www.who.int/tb/publications/2012/en/index.html. Accessed September 2013). Infection with this pathogen is via the inhalation of aerosols containing a small number of bacilli [1]. Once in the lung, bacilli can be phagocytosed by alveolar macrophages (MФ), in which they may survive intracellularly by arresting phagosome maturation and phagolysosomal fusion [1–4] until the destruction of MФ, which then allows the bacilli to infect new MФ and thus to perpetuate the infection. However, host immunity is sufficient to control *M. tuberculosis* in 90% of infected people thanks to a combination of early innate and subsequent adaptive responses, as indicated by the fact that only 10% of those infected develop active TB (Global tuberculosis report, 2012, World Health Organization, WHO. Available: http://www.who.int/tb/publications/2012/en/index.html. Accessed September 2013) [5].

MФ respond to *M. tuberculosis* infection through multiple interconnected mechanisms. They produce reactive oxygen and nitrogen intermediates [6–8] and a wide spectrum of inflammatory mediators. Moreover, they activate intracellular autophagy mechanisms, thereby suppressing intracellular mycobacterial survival through enhanced interaction between mycobacterial phagosomes and autophagosomes [9]. Autophagy is an evolutionally conserved process in cells for clearing abnormal proteins and organelles in a lysosome-dependent manner. At the molecular level, the sequential steps of autophagy involve a series of factors, such as activation of PI-3K hVPS34 through its interaction with Beclin 1. The best-defined autophagic marker is the microtubule-associated protein 1 (MAP1) light chain 3 (LC3). LC3 undergoes several modifications, among them C-terminal proteolysis, to form LC3-I, which is then modified into the phosphatidylethanolamine-conjugated form, LC3-II, which is incorporated into autophagosomal membranes [10–12].

Recent findings have determined that autophagy is also the end result of the anti-mycobacterial activity of vitamin D, or more specifically its active form 1,25-dihydroxyvitamin D3 (1,25D3) [13], which has long been known to activate a direct antimicrobial pathway in human MФ [14]. The 1,25D3-induced autophagic antimicrobial pathway involves the generation of the peptides cathelicidin and defensin B4 (DEF4B), which exert direct antimicrobial activity against *M. tuberculosis* [15,16]. This pathway also synergizes with other cellular responses, such as TLR activation. Indeed, TLR2/1 activation by mycobacterial components can also trigger the vitamin D-dependent induction of cathelicidin through the generation of IL15 [17], and in synergy with the IL-1β pathway, the induction of DEFB4 [18]. Moreover, the vitamin D pathway is also induced by two T-cell-mediated mechanisms, IFN-γ [19] and CD40 ligand [20], both part of the host adaptive immune response. 

In MФ, other mechanisms, such as activation of nuclear liver X receptors (LXRs), contribute to the control of *M. tuberculosis* infection [21]. LXRs are key regulators of MФ function because they control the transcriptional programs involved in lipid homeostasis. Their participation in antimycobacterial responses was demonstrated in a study that showed that mice deficient in both LXR isoforms, LXRα and LXRβ, were more susceptible to infection, developing higher bacterial burdens and showing an increase in the size and number of granulomatous lesions. In addition to the contribution of LXRs to lipid homeostasis, in the last few years several targets of LXR activation, among them AIM (Apoptosis Inhibitor of Macrophages), have been identified to be involved in the modulation of immune responses [22–25]. AIM, also named Soluble Protein alpha (Spα), CD5L, and Api-6, is a 40-kDa glycoprotein secreted by tissue MФ (spleen, lymph node, thymus, bone marrow, liver and fetal liver) [22,24]. It has been implicated in a broad spectrum of biological functions, mostly by preventing the apoptosis of MФ and other cell types [26,27]. By modulating the activity of MФ, it participates in the pathogenesis of several infectious and inflammatory processes [23,26–31]. In this regard, results from transgenic mice overexpressing AIM indicate that this molecule supports the survival and phagocytic activity of MФ in liver inflammatory lesions in fulminant hepatitis [29]. AIM has also been involved in atherosclerosis by facilitating MФ survival within atherosclerotic lesions [31]. Evidence of a potential pro-oncogenic role of mAIM arises from two studies in transgenic mice in which its overexpression induced lung adenocarcinoma [30,32]. More recently, it has been described that AIM is incorporated into adipocytes, thereby reducing the activity of cytosolic fatty acid synthase, which stimulates lipolysis, thus resulting in the induction of adipocyte inflammation in association with metabolic disorders subsequent to obesity [33–35]. 

Furthermore, AIM circulates in serum in relatively high amounts [36], and results of proteomic profiling highlight it as a putative serum biomarker for inflammatory conditions such as atopic dermatitis[37], Kawasaky disease [38], as well as liver cirrhosis [39–41]. It was also proposed that AIM contributes to the homeostasis of IgM, on the basis of its presence in IgM but not IgG or IgA serum fractions [42] and its direct interaction with IgM [36]. Moreover, AIM acts as a pattern recognition molecule of LPS and LTA on the surface of Gram-positive and -negative bacteria [43], and it influences the monocyte inflammatory response to LPS and LTA by inhibiting TNF-α secretion [43]. 

Here we studied whether the LXR-target gene AIM further contributes to host innate immunity by modulating key MФ responses to *M. tuberculosis*. Our results indicate that AIM expression peaks in the early phase of infection, thereby inducing the synthesis of vitamin D-dependent antimicrobial peptides and subsequent autophagy mechanisms that lead to mycobacterial killing. All together, our data support the notion that AIM enhances the mycobactericidal activity of MФ, thus actively participating in the innate response against *M. tuberculosis*.

## Materials and Methods

### Reagents and chemicals

PBS comprised 150 mM NaCl, 8 mM Na_2_HPO_4_, and 1.5 mM KH_2_PO_4_, pH7.4; TBS comprised 140 mM NaCl, and 50 mM Tris-HCl, pH 7.4. The Griess reagent contained 1% sulfanilamide, 2.5% phosphoric acid, and 0.1% *N*-(1-naphthyl) ethylenediamine dihydrochloride (Sigma-Aldrich, St Louis, MO, USA).

### Bacteria

*M. tuberculosis* H37Rv Pasteur strain (MTB) was grown in 250-mL PYREX bottles in a shaking incubator at 37°C and at 120 rpm in Middlebrook 7H9 broth (Becton Dickinson, BD, Madrid, Spain) supplemented with 0.2% glycerol, 0.5% albumin-dextrose catalase (BD) and 0.05% Tween 80. Bottle caps were left half open to allow unlimited O_2_ availability. Bacteria were grown to mid-log phase and stored at -70°C in 3-mL aliquots. For phagocytosis and autophagy experiments, bacteria were labeled with FITC (MTB-FITC) (Sigma-Aldrich) as follows: 4x10^7^ bacteria were incubated for 1 h at rt in 0.2M Na_2_CO_3_-NaHCO_3_ buffer (pH 9.5) (Merck Millipore, Darmstadt, Germany) containing 0.01% (w/v) FITC. They were then washed three times with PBS and resuspended in RPMI 10% FCS medium. The MTB-FITC were prepared in large volumes, aliquoted, frozen, and stored at -80°C for later use.

### Mice, infection and chemotherapy

An animal experiment was performed to evaluate the evolution of AIM presence and expression during *M. tuberculosis* infection *in vivo*. All the procedures were performed and approved by the Animal Experimentation Ethics Committee of the Hospital *Universitari Germans Trias i Pujol* (registered as B9900005) and also approved by the Dept. d’Agricultura, Ramaderia, Pesca, Alimentació i Medi Natural of the Catalan Government, in accordance with current national and European Union legislation regarding the protection of experimental animals (Law 1997 of the Catalan Government; Spanish *Real Decreto* 1201/2005; and the European 86/609/CEE; 91/628/CEE; 92/65/CEE and 90/425/CEE). 6–8-week-old specific-pathogen-free (spf) C57BL/6 female mice (Harlan Laboratories, Sant Feliu de Codines, Spain) were kept under controlled conditions in a P3 facility with access to sterile food and water *ad libitum*. Mice (n=3 to n=5 per time point) were infected with MTB through aerosol inoculation as described [44]. The animals were euthanized at weeks 3, 6, 16, 19 and 21 by isoflurane (inhalation excess), following a strict protocol to prevent unnecessary suffering. Lung and spleen samples were used to evaluate tissue bacillary load, by plating serial dilutions on Middlebrook 7H11 agar plates (BD Diagnostics, Spark, USA). The number of CFUs was counted after incubation for 21 days at 37°C, and the results are expressed as CFUs/mL. Mice were orally treated with Isoniazid (INH) plus rifampicin (RIF) (25 and 10 mg/kg, respectively) once a week from weeks 6 to 14 postinfection, as previously described [45].

### Serum samples and Western blot

Blood samples obtained from the euthanized animals were kept at 4°C for 8 h, and serum was obtained by centrifugation at 2500 xg, aliquoted, and kept at -20°C until required. The optimization of the conditions to detect mAIM in serum as well as the specificity of the assay is detailed in Protocol S1 and Figure S1. Mouse serum (1μL) was resolved in 8% SDS-polyacrylamide gels under non-reducing conditions, and proteins were transferred to nitrocellulose membranes membranes (Biorad, Laboratories, UK). The membranes were blocked with Starting Block TBS buffer (Pierce, Perbio Science, Rockford, IL, USA) for 1 h at rt, incubated overnight at 4°C with anti-mouse AIM biotinylated poAb (0.1 µg/mL, R&D Systems, Minneapolis, MN, USA) diluted in blocking buffer and probed with IRDye680Cw-conjugated streptavidin (LI-COR Biosciences, Lincoln, NE, USA) diluted in blocking buffer for 60 min at rt. Three 15-min washes between steps were performed with TBS-0.01% Tween 20. A pool of uninfected mice sera were used to determine basal levels of mouse AIM (mAIM) in Western blots, where recombinant mAIM (rmAIM) was used as a positive control (see Figure S1). Bound Ab was detected with an Odyssey Infrared Imager, and densitometric analysis was performed using the Odyssey V.3 software (LI-COR). Fold increase in mAIM concentration was determined using mAIM basal levels (pool of sera from uninfected mice), set as 1, as a reference. 

### Cells

The stable cell transfectants THP1-Vector and THP1-hAIM were cultured in RPMI 1640 supplemented with 10% FCS, 2 mM glutamine (Lonza, Basel, Switzerland), 250 μg/mL geneticin (Invitrogen, Paisley, UK), 100 U/mL penicillin and 100 μg/mL streptomycin (Sigma-Aldrich). Prior to experiments, cells were differentiated to macrophages (MФ) by incubation with 10 ng/mL of PMA (Sigma-Aldrich) in RPMI 10% FCS medium for 48 h. They were then washed with PBS, and medium was replaced with RPMI 10% FCS 24 h before the addition of bacteria. 

### *In vitro* infection of THP1 MФ

Frozen aliquoted MTB was centrifuged at 2000 xg for 20 min to remove the 7H9 Middlebrook broth. Pelleted bacilli were resuspended with RPMI 10% FCS and vortexed for 1 min. MФ monolayers were infected at the indicated multiplicities of infection (MOIs), and non-ingested bacilli were removed after 4 h by washing three times with PBS. RPMI 10% FCS medium was subsequently replenished. 

### Real-time quantitative PCR

 MФ (10^6^) were infected at MOI 0.1 as described above. Cells were washed with PBS, and RNA was isolated at the indicated time points using the QIAzol reagent (QIAGEN, Hilden, Germany) and purified with an RNeasy mini kit (QIAGEN), following the manufacturer’s instructions. Total RNA (0.5 µg) was reverse-transcribed using the Transcriptor First Strand cDNA Synthesis Kit (Roche, Mannheim, Germany). Then, 2 µl of each RT reaction was amplified in a LightCycler® 480 PCR system (Roche), using the KAPA SYBR Fast Master Mix (KAPA Biosystems, Woburn, MA, USA). Samples were incubated for an initial denaturation at 95 °C for 5 min, followed by 40 PCR cycles under the following conditions: 95 °C for 10 s, 60 °C for 20 s and 72 °C for 10s. All the primer pairs used are listed in Table 1. Gene expression values were normalized to the expression levels of human acidic ribosomal protein (HuPo) [46]. Fold induction was calculated using the levels of expression of each gene at time 0 (uninfected) in THP1-Vector cells as a reference. When indicated, cells were pre-incubated with 10 ng/ml of Interferon-γ (R&D Systems) 24 h before infection.

**Table 1:** List of primers used in this study.

	Forward primer (5’- 3’)	Reverse primer (5’- 3’)
hAIM	GACGAGAAGCAACCCTTCAG	CCCAGAGCAGAGGTTGTCTC
DEFB4	GGTGTTTTTGGTGGTATAGGCG	AGGGCAAAAGACTGGATGACA
cathelicidin	TGCCCAGGTCCTCAGCTAC	GTGACTGCTGTGTCGTCCT
Beclin 1	GGCTGAGAGACTGGATCAGG	CTGCGTCTGGGCATAACG
HuPo	GAGAACTGTTATGGGGCTAT	TTCAACTGGAGAGGCAAAGG

### Western blot analysis of cell lysates

MФ (10^6^) were infected at MOI 0.1 as described above. They were then washed in cold PBS and lysed in cell lysis buffer [20 mM Tris (pH 7.5) containing 150mM NaCl, 1 mM EDTA, 1 mM EGTA, 1% Triton-X100, 1 mM Na3VO4, 1 mM PMSF (all from Sigma-Aldrich) and complete protease inhibitor mixture tablets (Roche)] for 30 min at 4°C. Nuclei and cell debris were removed by centrifugation at 8000 xg for 15 min, and protein concentration was measured with the BCA protein assay kit (Pierce Perbio Science, Rockford, IL, USA), following the manufacturer’s instructions. Human AIM and LC3 expression was monitored by SDS-PAGE and Western blot analysis. 40-50 µg of cell lysate was resolved in 10% or 12% SDS polyacrylamide gels, respectively, under reducing conditions and electrophoretically transferred to nitrocellulose membranes (Bio-Rad Laboratories). These were then blocked with Starting Block TBS buffer (Pierce) for 1 h at rt and incubated overnight at 4°C with biotinylated anti-hAIM poAb (0.2 µg/mL, R&D Systems, Minneapolis, MN, USA), anti-LC3 poAb (2 µg/mL; Novus Biologicals, Littleton, CO, USA), anti β-tubulin mAb (0.5 µg/mL, Sigma-Aldrich), or anti β-actin mAb (0.5 µg/mL, Sigma-Aldrich) all diluted in blocking buffer. The membranes were subsequently incubated with IRDye680Cw-conjugated streptavidin (LI-COR Biosciences, Lincoln, NE, USA), or secondary antibodies (IRDye 800Cw-conjugated goat anti-mouse IgG or IRDye 680Cw-conjugated goat anti-rabbit IgG) (LI-COR Biosciences), diluted in blocking buffer for 60 min at rt. Three 15-min washes between steps were performed with TBS-0.01% Tween 20. Bound streptavidin or Ab was detected with an Odyssey Infrared Imager, and densitometric analysis was performed using the Odyssey V.3 software (LI-COR Biosciences). 

### Crystal violet staining

MФ (10^5^) were infected as described above at MOI 0.1, 1 or 10. Cells were washed with PBS at the indicated time points, fixed by incubation with 10% formamide (Sigma-Aldrich) and then stained with a 0.5% (w/v) crystal violet (Sigma-Aldrich) solution in 2% ethanol for 10 min. The plates were then rinsed three times with water and allowed to dry, and the dye was solubilized in 2% SDS (w/v) (MerckMillipore) for 30 min at rt. Absorbance at 590 nm was recorded on a Varioskan Flash microplate reader (ThermoFisher, Waltham, MA, USA). Viable cell numbers were calculated against a standard curve of known cell numbers. 

### Apoptosis

MФ (10^5^) were infected during different lengths of time at MOI 1. They were then removed from plates with accutase (PAA Laboratories, UK), washed twice in ice-cold PBS, and stained with (PE)-conjugated Annexin V and 7-aminoactinomycin D (7AAD), following the manufacturer´s instructions (BD). Cells were fixed in PBS containing 5% paraformaldehyde (Panreac, Castellar del Vallès, Catalonia, Spain) for 20 min and analyzed with a FACSCantoII instrument and FACSDiva software (BD). Apoptosis was expressed as the percentage of Annexin V-positive 7-AAD-negative cells.

### Foam cell quantification

MФ (10^5^) were infected as described above at MOI 0.1 for 24 h in RPMI 1% FCS medium and subsequently stained with Nile Red as follows. Cells were fixed in PBS containing 5% PFA (Panreac) for 20 min, incubated with a 1mM Nile Red solution (Molecular Probes, Life Technologies, NY, USA) in DMSO and extensively washed with cold PBS. Nile Red incorporation was analyzed using a Zeiss Axio Observer Z1 Inverted Microscope and AxioVision 4.8 software (Carl Zeiss MicroImaging, Jena, Germany) or quantified by flow cytometry on a FACSCalibur instrument using the CellQuest software (BD).

### IL-8 measurements

MФ (5x10^4^) were infected as described above at the indicated MOIs, and culture supernatants were collected 24 h postinfection. IL-8 production was measured with the IL-8 OptEIA ELISA kit, following the manufacturer´s instructions (BD). 

### Quantification of intracellular mycobacterial growth

MФ (10^6^) were infected as described above at MOI 0.1 or 1, and non-ingested bacilli were removed after 4 h by washing three times with PBS. Cells were either lysed with sterile water or left in culture medium for further 24 or 72 h, before being lysed. Cellular lysates were centrifuged at 2000 xg for 20 min and resuspended vigorously in sterile water. The number of intracellular bacilli was measured by plating serial dilutions of cell lysates on Middlebrook 7H11 agar plates (BD) and counting bacterial colony formation after 21 days of incubation at 37°C. 

### Phagocytosis assay

MФ (10^5^) were infected with MTB-FITC at MOI 40 during different lengths of time (30 min-4 h) at 37°C or for 4 h at 4°C. Incubation at 4°C was performed to measure extracellular attachment rather than internalization, since no uptake occurs at this temperature. MФ were then extensively washed with cold PBS and fixed with PBS containing 5% PFA (Panreac) for 30 min. The percentage of FITC-positive cells was determined by flow cytometry on a FACSCantoII instrument and using FACSDiva software (BD).

### NO and ROS measurements

MФ (0.5x10^4^) were infected as described above at MOI 0.1 and 1 during different lengths of time (4–120 h). NO production by MTB-infected MФ was determined by nitrite measurement in the culture supernatant by the Griess assay. Supernatants (100 µL) from THP1 cultures were added in triplicate to an equal volume of Griess reagent and incubated at rt for 10 min. To measure ROS production, cells were loaded with 10 µM dichloro-dihydroxy fluorescein diacetate (H2-DCF-DA) (Sigma-Aldrich) in PBS for 30 min at 37°C in the dark. They were then washed twice and resuspended in 100µl of PBS. Absorbance at 540 or 485 nm, for NO and ROS production, respectively, was measured using a Varioskan Flash microplate reader (ThermoFisher). Supernatant nitrite concentrations were calculated against a standard curve of known NaNO_2_ concentrations. Intracellular ROS levels were calculated as a percentage of the uninfected control (THP1-Vector cells), indicated as 100%.

### Confocal microscopy studies

MФ (5x10^4^) differentiated in Millicell EZ slides (MerckMillipore) were infected with MTB-FITC at MOI 5. When indicated, cells were pre-incubated with 1mM of 3-methyladenine (3-MA) (Sigma) 45min before infection. At 24 or 72 h post-infection, cells were washed with PBS, and medium was replaced with prewarmed RPMI containing 100 nM LysoTracker Red (Molecular probes), and cells were further incubated at 37°C for 1 h. They were then washed three times with PBS and fixed with PBS containing 5% PFA (Panreac) for 30 min. After three more washes with PBS, cells were stained with an anti-LC3 poAb (4 µg/mL; Novus Biologicals) in PBS containing 0.3% Triton X-100 and 10% Human AB serum (Sigma) for 24h at 4°C. Then, an Alexa Fluor® 647 labeled F(ab')_2_ Fragment of Goat Anti-Rabbit IgG (H+L) secondary antibody (2 µg/mL, Invitrogen) was incubated for 1 h at rt in PBS containing 0.3% Triton X-100. Between steps, unbound antibody was removed with three washes with PBS. Finally, nuclei were stained with PBS containing 800nM Hoechst solution (Invitrogen) for 10 min at rt. Cells were washed three times with PBS, and coverslips were mounted in Fluoromount mounting media (Sigma-Aldrich) and left at 4°C overnight. The slides were examined using a FluoView™ FV1000 Spectral Confocal microscope and analyzed with FluoView™ FV10-ASW 3.1 software (Olympus, Shinjuku, Tokyo, Japan). The percentage of lysosome-MTB as well as lysosome-MTB-LC3 co-localization was calculated by counting the overlapping of fluorescence in random fields for a minimum of 200 internalized bacilli for three independent infections. LC3 puncta per cell was determined using the Image J software and puncta analyzer plug-in (NIH, Maryland, US), in thresholded images with size from 5 to 20 pixel^2^ and puncta circularity 0.8-1 as described previously [47], in random fields for a minimum of 100 cells for three independent infections.

### Statistical analysis

Data are presented as mean ± SEM of at least 3 experiments. Statistical analysis was performed with Graphpad Prism V.5 software using the Student’s t test, and one-way or two-way ANOVA. Values of p≤0.05 were considered significant.

## Results

### AIM levels increase dramatically in mouse serum after *M. tuberculosis* infection

**Figure 1:**
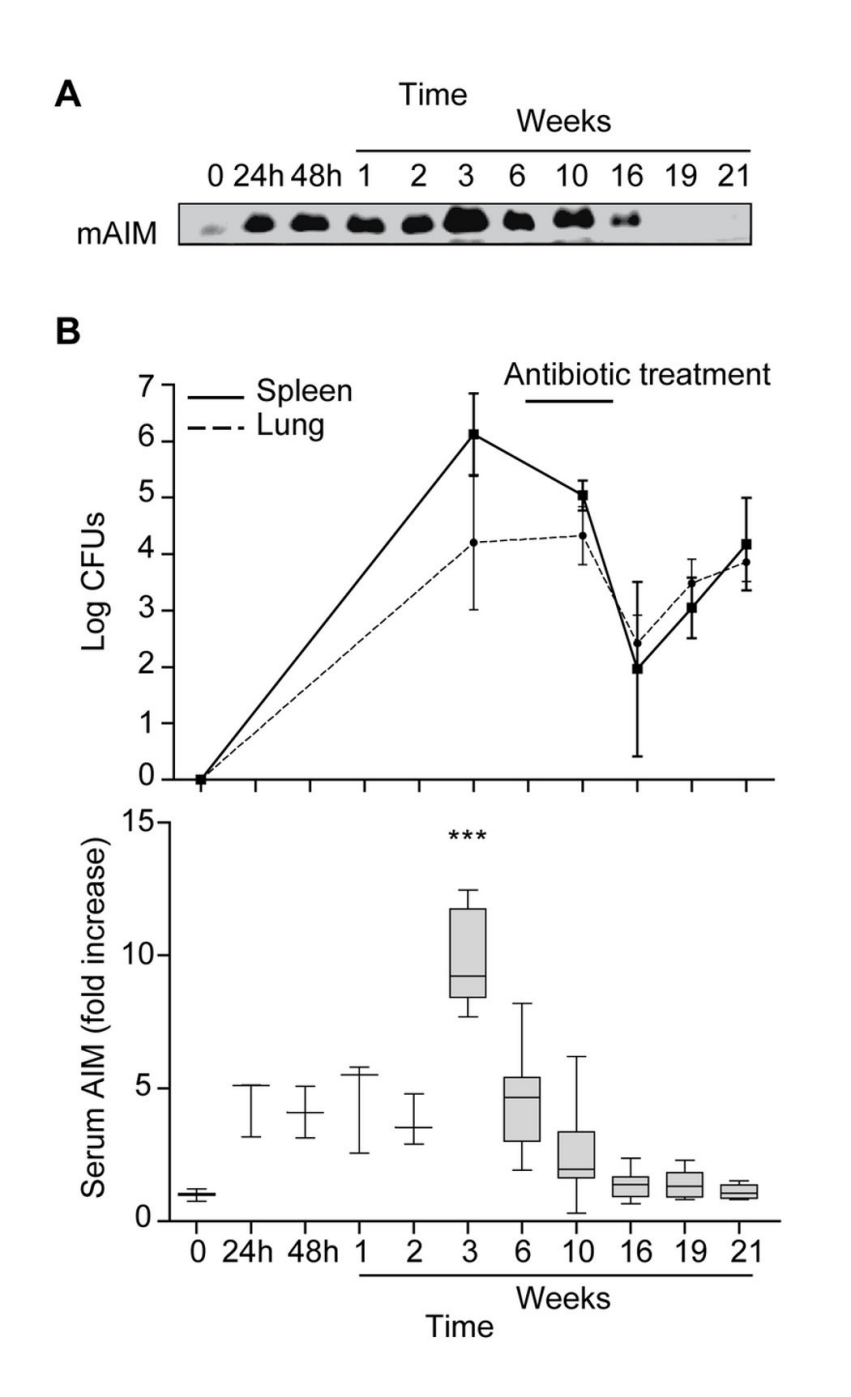
*M. tuberculosis* increases mAIM serum levels in an *in* *vivo* infection model. C57BL/6 mice were infected with *M. tuberculosis* H37Rv through aerosol inoculation. Mice were treated with INH/RIF for 8 weeks (w6 to w14) at which point antibiotic was withdrawn, and infection was allowed to reactivate. mAIM serum levels and bacillary load in the lung and spleen were measured at several time points post-infection (24 h - 21 weeks). A) Representative image of mAIM levels analyzed by Western blot of serum samples. B) Graphs showing spleen and lung bacterial loads at the indicated times (upper graph) and mAIM protein intensity (lower graph) data. Box plots show median values and 5-95 percentile values, from 1µl serum (n=3 to n=5). Fold induction levels were calculated using as reference the serum mAIM from a pool of 5 C57BL/6 uninfected healthy animals, set as 1. *p≤0.05; **p≤0.01; ***p≤0.001 two-way ANOVA.

Both human (hAIM) and mAIM have been detected circulating in serum [22,36]. Our first goal was to analyze whether, like several LXR and RXR target genes, AIM expression in MФ is induced in response to *M. tuberculosis* infection [21]. We examined the concentrations of serum mAIM in an experimental model of *M. tuberculosis* infection in order to determine an *in vivo* biological link between this protein and the disease (Figure 1). Mice were infected with MTB by aerosol inoculation, and lung and spleen bacterial load, as well as serum mAIM were analyzed at several times post-infection. mAIM serum detection by Western blot was optimized as shown in Figure S1. Figure 1A shows a representative Western blot analysis of serum mAIM levels. The graph depicting results from 3 to 5 mice per time point in Figure 1B shows that mAIM levels increased 5-fold immediately after infection and remained constant for 2 weeks. A second peak of this protein was detected at week 3 post-infection, reaching maximum levels (10-fold those of uninfected mice). This peak coincided with maximum CFU counts in lung and spleen. Concentrations of mAIM dropped to basal levels thereafter and during antibiotic treatment. Reactivation of the infection by antibiotic withdrawal did not affect serum mAIM levels, which remained constant for the rest of the experiment.

### hAIM expression increases the survival of *M. tuberculosis*-infected THP1 MФ

**Figure 2:**
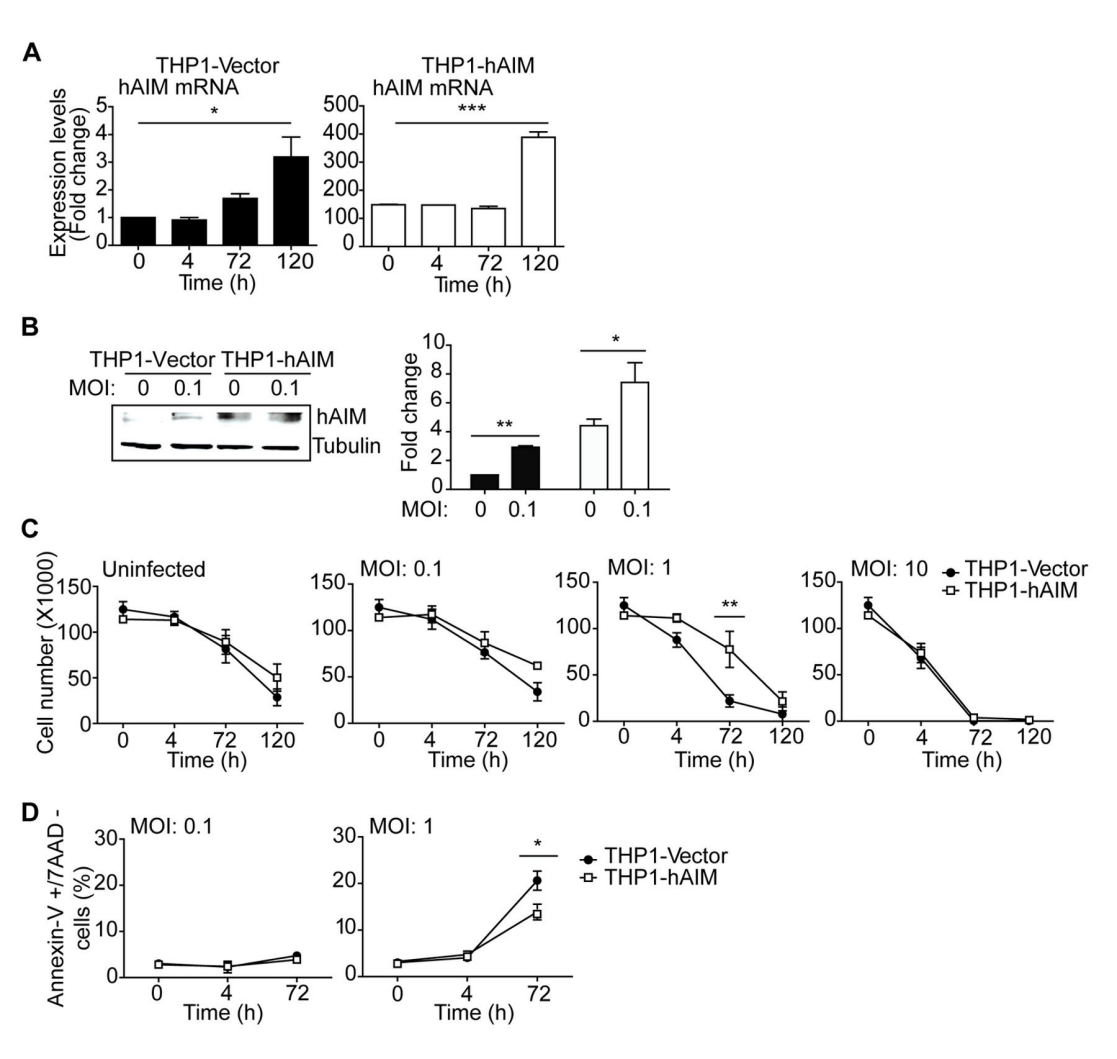
Expression of hAIM infection increases the survival of *M. tuberculosis*-infected MФ. Stably transfected THP1-Vector (control) and THP1-hAIM MФ were infected with *M. tuberculosis* at the indicated MOIs, and hAIM expression and cell viability were analyzed at the indicated time points. A) Mean ± SEM mRNA of hAIM expression were determined by RT-qPCR on cells infected at MOI 0.1. hAIM mRNA values are represented as fold change vs. uninfected THP1-Vector cells, set as 1. *p≤0.05; ***p≤0.001; one-way ANOVA. B) Western blot analysis of hAIM protein levels in cell lysates at MOI 0.1 and 0 to 120 h post-infection. Equal loading was determined by probing against tubulin. Left panel, western blot; right panel, fold induction levels, which were calculated by setting the background signal of uninfected THP1-Vector cells to 1 as a reference. C) The number of viable cells was determined by crystal violet staining and quantified using a standard curve of known input cell numbers. D) Apoptosis was assessed using Annexin V-7AAD staining and analyzed by flow cytometry. Results are expressed as percentage of Annexin V-positive, 7AAD-negative cells. All graphs are from three independent experiments performed in triplicate. *p≤0.05; **p≤0.01; two-way ANOVA.

Cultured MФ, in contrast to tissue MФ, do not express AIM [25,26]. To overcome this limitation, we generated a MФ cell line stably expressing hAIM (THP1-hAIM) (unpublished data). Our first goal was to analyze whether AIM mRNA and protein expression is induced in the THP1 cell line *in vitro* in response to *M. tuberculosis* infection [21]. MTB infection at MOI 0.1 induced hAIM mRNA synthesis in THP1-Vector (control) and THP1-hAIM cell lines, although the increase was significant 120 h post-infection (Figure 2A). At this time point, hAIM protein was also expressed in both cell lines, albeit the levels were higher in THP1-hAIM cells (Figure 2B). 

To assess whether the anti-apoptotic role of AIM [23,25,29,31,48] is conserved in *M. tuberculosis* infection, stable THP1 cell transfectants were infected at three MOIs, and cell viability was assessed by crystal violet staining. *M. tuberculosis* infection affected MФ viability in a MOI- and time-dependent manner (Figure 2C). The data further show that the numbers of uninfected THP1-hAIM cells were similar to those of THP1-Vector cells over time. Interestingly, infection at low MOI (0.1) did not significantly change THP1 cell survival when compared to that of uninfected cells. However, increasing the MOI to 1 resulted in higher THP1-Vector cell death, which was significantly greater than that observed in THP1-hAIM cells: at day 3 post-infection, the number of viable THP1-hAIM cells was double than that of THP1-Vector cells (7x10^4^ vs. 3x10^4^). Longer infection times (5 days) or increasing the MOI to 10 almost totally compromised cell viability. The dynamics of such infection time involve continuous uptake, killing of some bacteria, and too many organisms being internalized by MФ, and therefore most of the subsequent experiments were performed in shorter lengths of time (up to 72h). The data suggest that expression of hAIM contributes to the survival of *M. tuberculosis*-infected MФ. In accordance, hAIM conferred MФ resistance to *M. tuberculosis*-induced apoptosis, as measured by Annexin V and 7AAD staining. In these assays, no apoptosis was detected at MOI 0.1, while the percentage of apoptotic cells (Annexin V+, 7AAD- cells) was significantly lower in THP1-hAIM cells at MOI 1 (Figure 2D). These data strengthen the notion that hAIM supports MФ survival in the setting of *M. tuberculosis* infection.

### hAIM enhances MФ foam cell formation and IL-8 secretion

**Figure 3:**
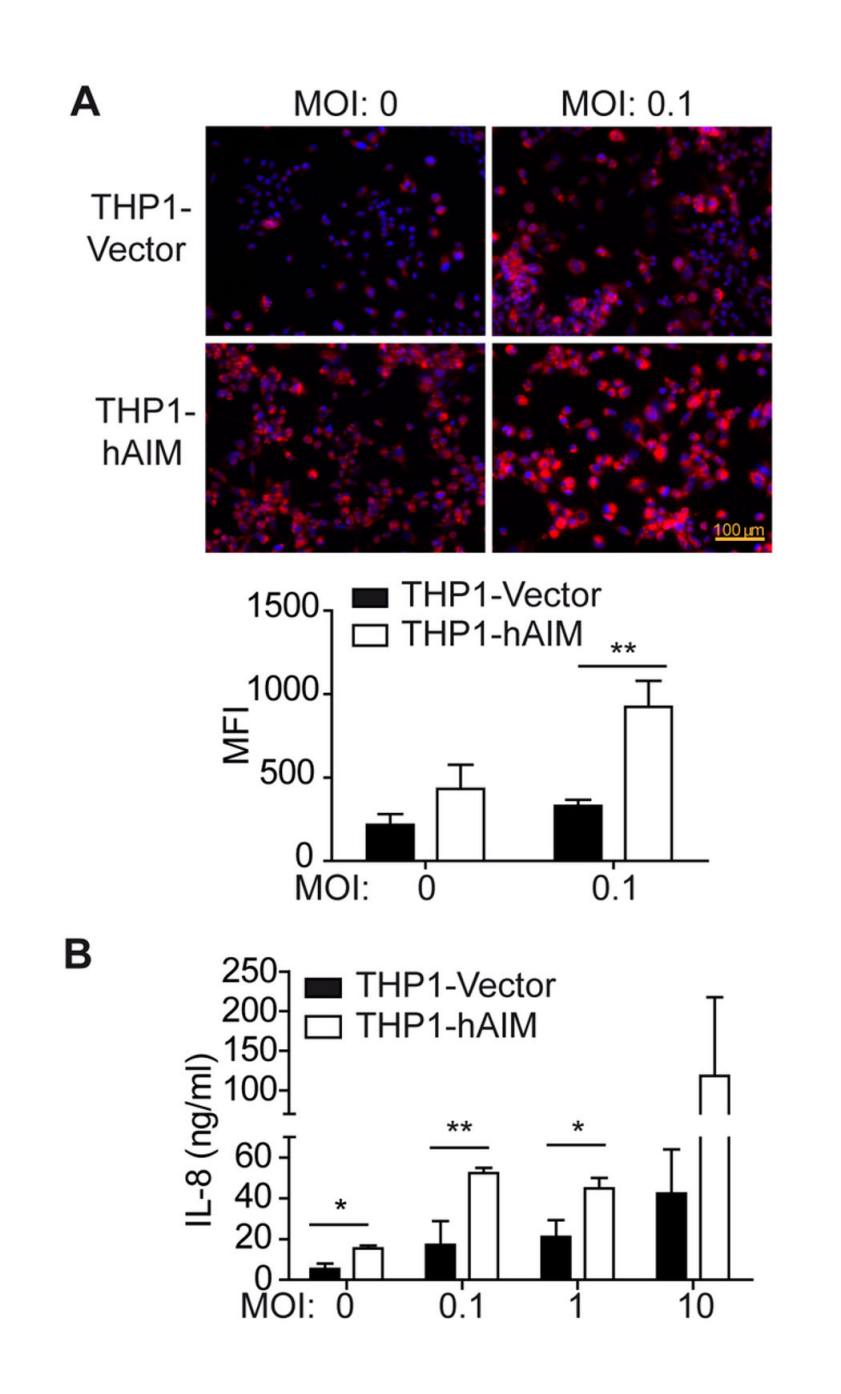
Human AIM enhances foam cell formation and IL-8 secretion. A) THP1 MФ were infected with *M. tuberculosis* at MOI 0.1 for 24 h in RPMI 1% FBS medium, fixed, stained with Nile Red and observed by fluorescent microscopy (left panel), or quantified by flow cytometry (right graphic). MFI, Median Fluorescence Intensity. B) The amount of IL-8 in culture supernatants from *M. tuberculosis*-infected THP1 MФ at the indicated MOIs during 24 h was determined by ELISA. Mean ± SEM from three independent experiments performed in triplicate are shown. *p≤0.05; **p≤0.01; two-way ANOVA.

MФ foam cell formation caused by intracellular lipid accumulation is a hallmark of *M. tuberculosis* infection [49,50]. We have recently observed that in atherosclerosis hAIM increases foam cell formation induced by modified lipoproteins (namely oxLDL) (unpublished data). We therefore tested whether hAIM modifies *M. tuberculosis*-induced MФ lipid accumulation. For this purpose, we stained infected MФ with the lipid specific dye Nile Red. *M. tuberculosis* infection increased foam cell formation in THP1-Vector cells, and this formation was enhanced in hAIM-transfected cells (upper panel), as shown by the fluorescence microscopy analysis in Figure 3A. Quantification by flow cytometry analysis (lower graph) indicated that the lipid content of THP1-hAIM cells reached ~ 4-fold that of control THP1-Vector cells. 

Infected LXR-deficient mice show decreased pulmonary neutrophilia [21]. We analyzed whether AIM contributes to *M. tuberculosis*-infected MФ secretion of the chemokine IL-8, a highly attractant molecule for neutrophils [51]. Indeed, we found that the expression of hAIM increased MФ IL-8 production by ~3-fold (Figure 3B). All together, these data indicate that hAIM contributes to infected MФ foam cell formation as well as to IL-8 secretion.

### Expression of hAIM reduces MФ mycobacterial load

**Figure 4:**
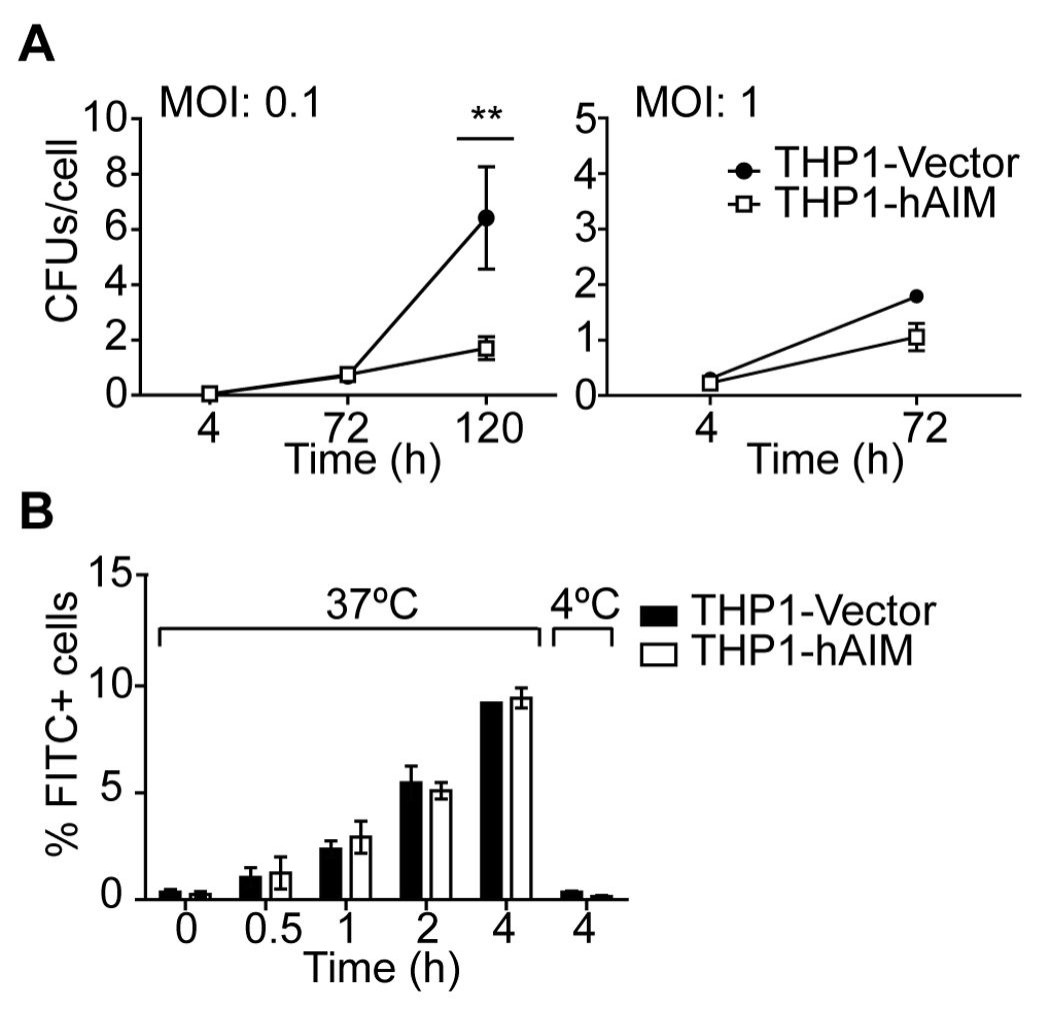
hAIM increases the intracellular killing of *M. tuberculosis* without modifying the phagocytic capacity of MФ. A) Stably transfected THP1 MФ were infected with *M. tuberculosis* at MOI 0.1 and 1. Four, 72 and 120 h later, cells were lysed and intracellular CFU numbers were determined by serial dilutions on 7h9 agar plates. CFUs per cell were calculated by dividing CFUs by number of viable cells determined by crystal violet staining at each time point. Mean ± SEM from three independent experiments performed in duplicate. **p≤0.01; *p≤0.05 two-way ANOVA. B) THP1 MФ were incubated with FITC-labelled bacilli at MOI 40 and the percentage of FITC-positive cells at the indicated time points and temperature was determined by flow cytometry. Results are expressed as the % of FITC-positive cells at each time point and show the mean ± SEM from three independent experiments.

Using a colony forming unit (CFU) assay, we tested whether hAIM participates in MФ mycobactericidal activity. Expression of hAIM significantly reduced the number of viable bacilli per cell, with ~70% of the bacteria being killed at day 5 post-infection at MOI 0.1, and ~50% at day 3 post-infection at MOI 1 (Figure 4A). Given that infection rate and time affected cell viability (Figure 2B), CFUs per cell were calculated by dividing CFUs by number of viable cells, as determined by staining with the vital dye crystal violet. To discard the possibility that reduced bacterial load in hAIM-expressing cells was due to decreased initial phagocytosis, bacilli were fluorescently labeled with FITC, and THP1 MФ bacterial uptake was analyzed by flow cytometry (Figure 4B). In these assays, the percentage of FITC-positive cells increased over time when the experiments were performed at 37°C, but not at 4°C. These observations thus indicate that increases in fluorescence were due to uptake rather than to bacterial adherence to the cell surface. Nevertheless, no differences were detected between THP1-Vector and THP1-hAIM cells, thereby suggesting no participation of hAIM in bacterial uptake. In summary, our data indicate that hAIM plays a crucial role in enhancing mycobactericidal responses in MФ. 

### hAIM modulates the MФ production of radical oxygen species

**Figure 5:**
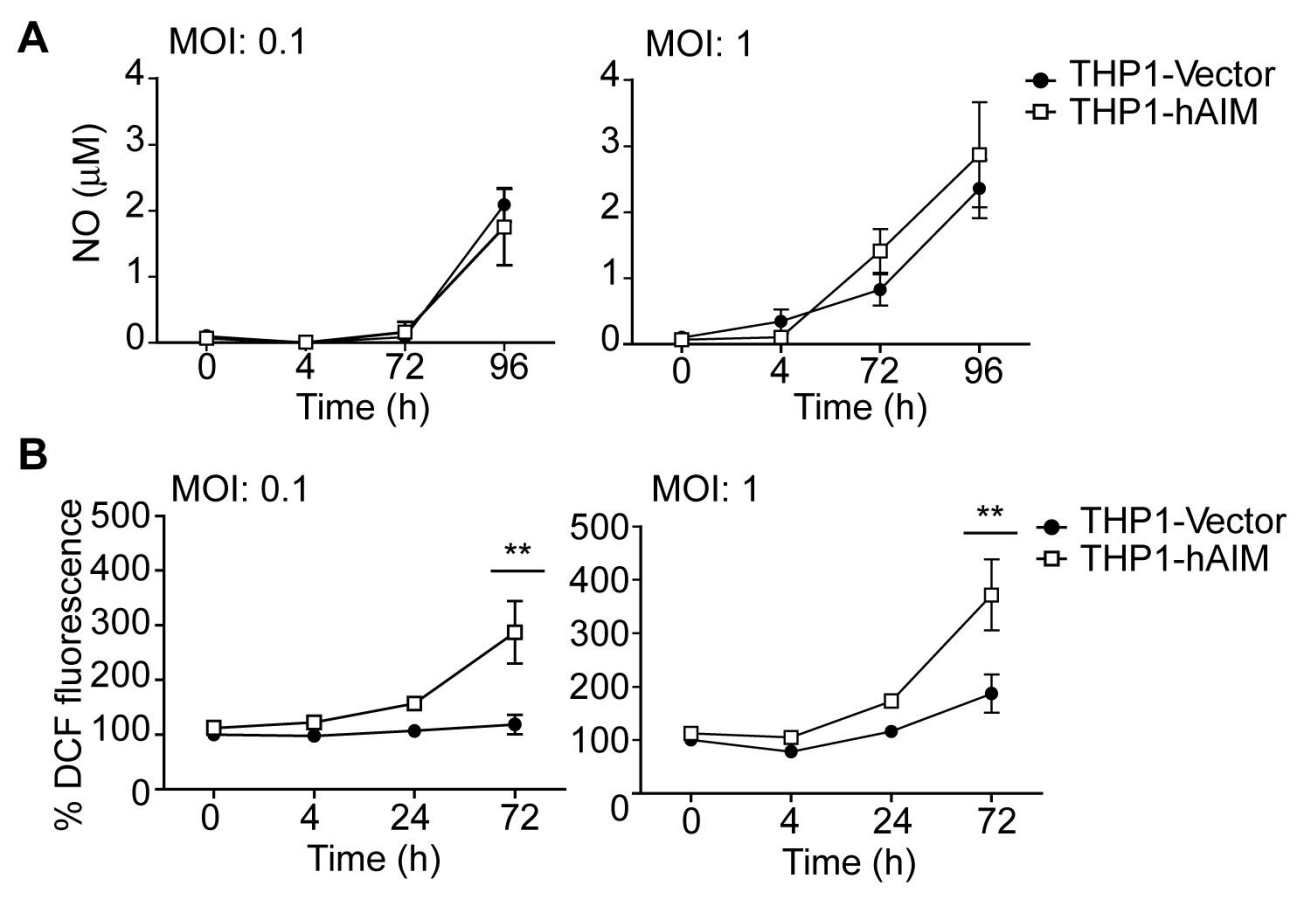
Effects of hAIM on NO and ROS production. THP1 MФ were infected with *M. tuberculosis* at MOI 0.1 and 1 during the times indicated, and the production of NO and ROS was determined as follows. A) Nitrite levels were measured in the supernatants using the Griess reagent, and values were calculated against a standard curve of known NaNO_2_ concentrations. B) Intracellular ROS release was quantified via the changes of DCF fluorescence. ROS levels were calculated as a percentage of the uninfected control (THP1-Vector cells), indicated as 100%. Mean ± SEM from three independent experiments, performed in triplicate are shown. *p≤0.05; **p≤0.01; two-way ANOVA.

Our next set of experiments analyzed whether the hAIM-mediated mycobactericidal effect was due to increased MФ production of NO or reactive oxygen species (ROS). NO levels in the supernatants as well as intracellular ROS in infected MФ were analyzed by the Griess method and by H2-DCF-DA-induced fluorescence (Figure 5A and B, respectively). Although *M. tuberculosis* infection induced NO secretion in both cell lines in a time- and MOI-dependent manner, NO production was very low (0-2µM) and did not differ between THP1-Vector and THP1-hAIM cells (Figure 5A). In fact, it is well known that NO production by human MФ is not as high as that of murine MФ [52] and that the involvement of this process as a microbactericidal mechanism in humans is controversial [7]. Conversely, upon infection, a time- and dose-dependent significant rise in ROS production was observed in both cell lines. This increase was further intensified by the expression of hAIM (Figure 5B). Therefore, our data indicate that hAIM induces increased ROS production in infected MФ.

### hAIM upregulates the expression of the antimicrobial peptides Defensin 4B (DEF4B) and cathelicidin

**Figure 6:**
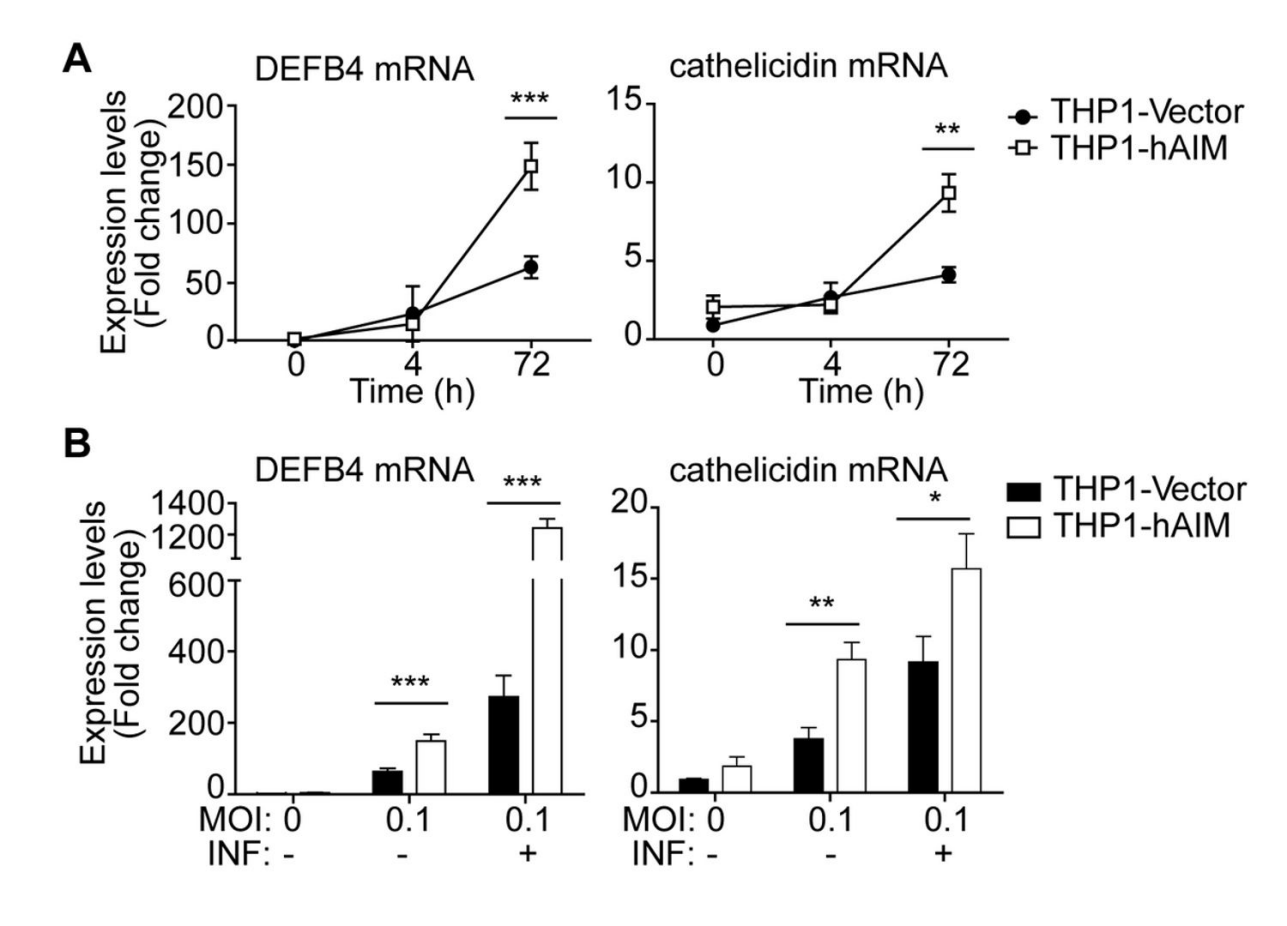
hAIM increases the expression of the antimicrobial peptides DEFB4 and cathelicidin. A) Stably transfected THP1 MФ were infected with *M. tuberculosis* at MOI 0.1, and mRNA levels of DEFB4 and cathelicidin were determined by RT-qPCR. B) The same experiment was performed but cells were incubated with rIFNγ (10 ng/ml) 24 h prior to infection. mRNA mean fold change values is relative to uninfected THP1-Vector ± SEM, set as 1, from three independent experiments. *p≤0.05; **p≤0.01; ***p≤0.001 two-way ANOVA.

 We next studied whether expression of hAIM modulates the induction of DEF4B and cathelicidin (LL-37) —two antimicrobial peptides of the vitamin D-dependent antimicrobial pathway— in infected MФ. Interestingly, *M. tuberculosis* infection induced MФ synthesis of DEF4B and cathelicidin mRNA at 72 h postinfection, and this was enhanced ~2- fold in hAIM-expressing cells (Figure 6A). We next tested whether hAIM was able to modulate IFN-γ-induced anti-microbial responses. In this regard, the expression of hAIM synergized with IFN-γ in further increasing the gene expression of DEF4B ~2-fold, but not that of cathelicidin (Figure 6B). 

### hAIM expression activates autophagy-dependent microbicidal mechanisms

**Figure 7:**
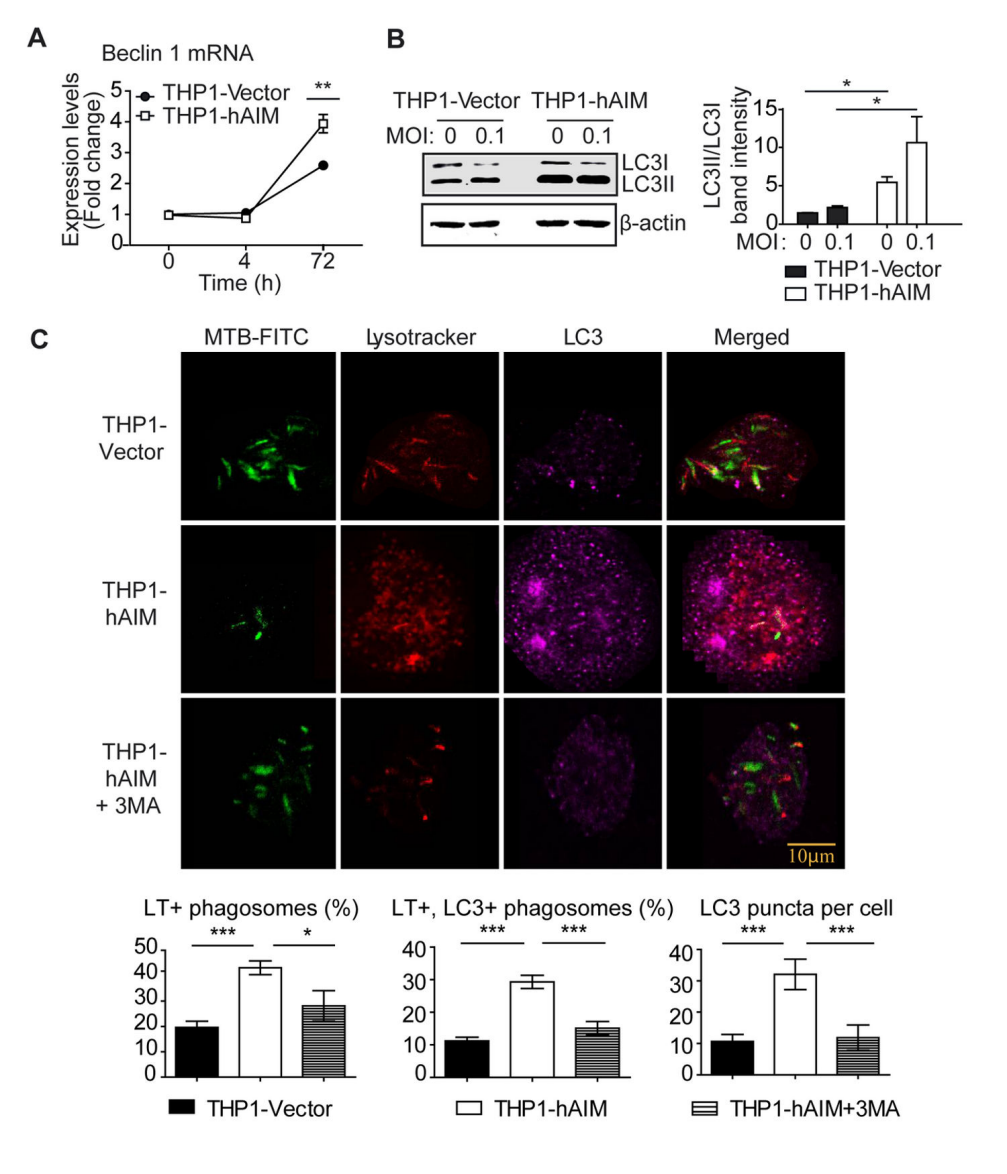
hAIM promotes autophagy and endosome-lysosome fusion in *M. tuberculosis*-infected THP1 cells. Stably transfected THP1 MФ were infected with *M. tuberculosis* H37Rv at MOI 0.1, and autophagy was analyzed as follows. A) mRNA levels of autophagy related protein Beclin 1 were determined by RT-qPCR at the indicated times. mRNA mean fold change values are relative to uninfected THP1-Vector ± SEM, set as 1, from three independent experiments. *p≤0.05; **p≤0.01; ***p≤0.001 two-way ANOVA. B) LC3 expression was analyzed by Western blot in cell lysates of uninfected and *M. tuberculosis*-infected THP MФ for 24 h. Left: representative Western blot image. Right: protein signal intensities were quantified and plotted as LC3-II/LC3-I ratio after normalization to the control protein actin. Mean ± SEM of three independent experiments. *p≤0.05; **p≤0.01; ***p≤0.001, one-way ANOVA. C) *M. tuberculosis*-lysosome co-localization analysis. Upper panel: representative confocal microscopy images showing co-localization of FITC-labeled *M. tuberculosis* bacilli (green), cellular lysosomes (red) and LC3 (purple) 72 h post-infection, in the presence or absence of the autophagy inhibitor 3-MA. Lysosomes were stained with LysoTracker Red, and LC3 with a specific antibody. Lower panel: mean ± SEM quantitative data show *M. tuberculosis*-lysosome, *M. tuberculosis*-lysosome-LC3 co-localization and LC3 puncta per cell in three independent experiments, with each experiment including at least 200 internalized bacilli or 100 cells scored in random fields. *p≤0.05; **p≤0.01; ***p≤0.001, one-way ANOVA.

The vitamin D-antimicrobial pathway controls autophagy and phagosome maturation [53]. We therefore analyzed whether hAIM affects this pathway by modulating the expression of the autophagosome protein Beclin 1. In this regard, the mRNA of this gene increased in THP1-Vector cells at 72 h post-infection, and the expression of hAIM further increased Beclin 1 mRNA levels 1.5-fold (Figure 7A). Moreover, to study autophagosome formation, we used Western blots to quantify the content of LC3-II and LC3-I [54] in cellular lysates. *M. tuberculosis* infection slightly increased the LC3II / LC3 I ratio in THP1-Vector MФ (Figure 7B). Interestingly, hAIM expression enhanced this ratio 5-fold, thereby suggesting increased autophagosome formation. We next tested whether hAIM enhances the acidification of mycobacterial phagosomes and whether this was due to autophagy-dependent mechanisms by analyzing the co-localization of LC3, as well as the number of LC3 puncta per cell. THP1 MФ were infected with FITC-labeled bacilli for 24 (data not shown) and 72 h and stained with LysoTracker, an acidotropic fluorescent dye that accumulates in acidic organelles, as well as an antibody against LC3. No differences between the two cell lines were observed at 24 h postinfection regarding phagosomal acidification (data not shown). However, hAIM-expressing cells showed 43.2 % ± 16.4 FITC-bacteria co-localization with LysoTracker vs 19.6 ± 12.5 in THP1-Vector cells (p 0.0029 Student t test) at 72 h postinfection (Figure 7C). These findings show that hAIM expression in MФ renders mycobacterial phagosomes more susceptible to acidification. Furthermore, this was coincident with an increase of LC3 co-localization with the bacterial-containing phagosomes in hAIM-expressing cells (32.7% ± 22) versus THP1-Vector cells (10.7% ± 10) (p 0.0004 Student t test). We further analyzed the amount of LC3 puncta in infected cells at this time point as a measure of autophagosome formation, and found that hAIM-expressing cells almost triplicated the LC3 puncta per cell as compared to THP1-Vector cells (29.3± 20 vs 11.2 ± 11, p <0.0001 Student t test). Interestingly, addition of the autophagy blocker 3-MA inhibited these effects, further suggesting a contribution of hAIM to autophagy. All together, our results support the notion that hAIM contributes to increasing MФ autophagosome formation during *M. tuberculosis* infection.

## Discussion

Here we demonstrate that hAIM participates in several key aspects of MФ response to *M. tuberculosis*. This finding is of relevance because in previous studies we showed that hAIM is involved in pattern recognition of bacteria and in the modulation of monocyte inflammatory responses [43]. The present study now reveals that the participation of hAIM in innate immunity goes beyond these activities. Our data support the notion that hAIM makes a relevant contribution to the MФ autophagy mechanisms that lead to intracellular mycobacterial killing.

The involvement of AIM in the initial innate immune response to *M. tuberculosis* infection is illustrated by the observation that 24 h after infection its serum levels increased 5-fold and peaked at 10-fold at 3 weeks, during the exponential growth of the bacilli [55]. At this point, when bacterial growth control by the adaptive immune response takes place, AIM serum levels dropped to almost basal levels. Antibiotic treatment or reactivation by antibiotic removal did not result in a second peak of serum AIM. This experiment lacks the control group of uninfected mice, and conclusions should be considered with caution. However, the data reinforce the notion that hAIM is involved in the initial inflammatory burst of the host response to infection. It also suggests that a high threshold of bacterial load and subsequent inflammation are needed for hAIM to increase its plasma levels. Our results are of relevance because the protein AIM is composed exclusively of Scavenger Receptor Cysteine-Rich (SRCR) domains. Each of these domains consists of ~100 amino acids containing 6–8 cysteine residues with a well conserved disulfide bond pattern [56]. The SRCR domain is present in proteins that contribute to the immune defence *M. tuberculosis* infection, such as macrophage SR-AI [57] and Macrophage Receptor with Collagenous Structure (MARCO) [58]. Moreover, genetic variations of MARCO have been associated with susceptibility to pulmonary tuberculosis in a Gambian population [59]. It is interesting that the ectodomain of CD163 (sCD163), another member of the SRCR protein family expressed by MФ, has been found to be elevated in serum of TB patients [60]. In that study increased pre-treatment serum levels of sCD163 appeared to be an independent predictor of mortality during treatment, as well as of long-term mortality in verified cases of TB from Guinea-Bissau [60]. These observations open up the possibility that related SRCR proteins are predictors of TB disease in humans. 

In our effort to decipher the functional involvement of hAIM in *M. tuberculosis* infection of MФ, we performed a range of *in vitro* experiments. Our initial studies confirmed that, like other LXR target genes such as ApoE and ABCA1 [21], AIM MФ mRNA expression was induced in response to infection. This finding is relevant because MФ expression of AIM is tightly regulated. In this regard, low levels of hAIM mRNA and null protein expression were detected by real-time PCR and Western blot, respectively, in differentiated THP1 cells (unpublished data). In our study, due to stable transfection of the cDNA encoding hAIM in these cells, the levels of hAIM mRNA were higher in THP1-hAIM cells over time. The data indicate that the upregulation of hAIM expression in THP1-Vector cells occurred later than the observed increase in anti-mycobacterial activity in THP1-hAIM cells.

We used the THP1 cell line because THP1-PMA differentiated MФ have been demonstrated to be a suitable cellular model for *M. tuberculosis* infection [61,62], including the study of the vitamin D antimicrobial pathway [16]. Using this cell line, we assessed whether the anti-apoptotic activity of AIM is conserved in response to *M. tuberculosis* infection. Indeed, THP1-hAIM cells were more resistant to infection-induced cell death and apoptosis, thereby confirming a pro-survival role for this protein in these settings. These results also served to determine that, under our experimental conditions, *M. tuberculosis* did not affect THP1 cell survival when infected at MOI 0.1. Therefore, our studies at MOI 0.1 helped to decipher the contribution of hAIM to MФ responses, independent of its anti-apoptotic effects. 

We also observed that hAIM expression enhanced foam cell formation both in uninfected and infected cells, thus conferring a role for the human form of this protein in MФ lipid accumulation. This finding contrasts with previous results, in which *M. tuberculosis*-infected LXR-deficient mice showed enhanced foam cell formation [21]. This apparent contradiction is consistent with our own observations, which point to a distinct role of human and mouse AIM in MФ lipid accumulation (unpublished data). In the context of atherosclerosis, assessment of oxLDL foam cell formation evidenced a new role for hAIM, which enhances MФ lipid storage through increased uptake (unpublished data). This enhancement is not conserved in mAIM [31]. Foamy MФ are key participants in both sustaining persistent bacteria and contributing to the tissue pathology, which might lead to cavitation and the release of infectious bacilli [49,50]. The lipid may serve as a source of nutrients for the pathogen, enabling its survival within the cell. On the other hand, lipids play multiple roles as determinants of phagosomal formation and fate and as coordinators of the recruitment and retention of key phagocytic proteins [63,64]. The participation of hAIM in *M. tuberculosis*-induced foam cell formation and its specific consequences deserves further study.

Given that LXR-deficient mice fail to mount an effective early neutrophilic airway response to infection [21] and that THP1-hAIM-expressing cells showed increased secretion of IL-8, a highly chemoattractant chemokine for neutrophils, we hypothesize that AIM contributes to neutrophil attraction. Furthermore, our studies provide strong evidence that this protein participates in the intracellular mycobactericidal activity of MФ. Functional studies suggest that this activity is mediated through enhanced ROS secretion and autophagy mechanisms in MФ. The expression of hAIM induced an increase in the transcription of the antimicrobial peptides DEF4B and cathelicidin in *M. tuberculosis*-infected MФ. Given that hAIM expression synergized with IFN-γ in further increasing DEF4B mRNA levels ~2 fold, but not those of cathelicidin, our results suggest that, in addition to the vitamin D pathway, hAIM also activates the IL-1β pathway of DEFB4 production [18]. hAIM-enhanced cathelicidin and DEFB4 production was concomitant with increased transcription of the autophagy-related gene Beclin 1. Consequently, we observed enhanced flux of bacteria through phagosomes to phagolysosomes as evidenced by the significantly higher number of bacilli localized in the phagolysosomal compartments. Several additional evidences point to a role for hAIM in enhancing autophagy mechanisms. Its expression induced enhanced co-localization of this protein with the bacterial-containing autophagolysosomes. The elevated LC3 puncta in hAIM-expressing cells further suggest a contribution of hAIM to autophagy. Moreover, these effects were inhibited by the autophagy blocker 3-MA [65]. Enhanced LC3 co-localization could also reflect an increase of LC3-associated phagocytosis (LAP) [66] by hAIM, rather than autophagy. Electronic microscope image analysis would have confirmed whether bacteria are in double membrane, autophagosomal compartments [66]. However, our data is supported by previous work that has shown that *M. tuberculosis* can be subject to autophagy [9,53,67,68]. Therefore, our work suggests that overexpression of hAIM has many interconnected effects on the THP-1 MФ, ranging from decreased apoptosis to increased foam cell formation. They also include enhanced IL-8 secretion. Moreover, the boosting of ROS production and antimicrobial peptide synthesis, concomitant with an increase in autophagy mechanisms, could explain enhanced mycobactericidal capacity of hAIM-expressing MФ. 

To our knowledge, AIM is the first protein belonging to the SRCR family to be proven to induce autophagy in response to *M. tuberculosis*. The high degree of structural and phylogenetic conservation of the SRCR domains has helped elucidate several common functions among SRCR proteins, such as binding to bacteria [56,69–72]. The finding that hAIM participates in the vitamin D antimicrobial pathway opens up the possibility that other SRCR-containing proteins act in a similar way.

In summary, our *in vivo* observations and *in vitro* results indicate that hAIM is a key orchestrator of MФ bactericidal responses to *M. tuberculosis*.

## Supporting Information

Figure S1 **Optimization of mAIM detection in serum by western blot analysis.** A) Representative image of mAIM detection analyzed by Western blot of serum samples. Either rmAIM or the indicated amounts of serum were resolved in 8% SDS polyacrilamide gels under R or NR conditions and the presence of mAIM was detected with an specific antibody. B) Graph depicting the results of the densitometric analysis performed using the Odyssey V.3 software (LI-COR).
(TIF)

Protocol S1 (DOCX)
