# Supplementary figures and images for: The Scavenger Protein Apoptosis Inhibitor of Macrophages (AIM) Potentiates the Antimicrobial Response against Mycobacterium tuberculosis by Enhancing Autophagy
Source: PLoS One. 2013 Nov 4;8(11):e79670. doi: 10.1371/journal.pone.0079670 (PMC3817138; doi:10.1371/journal.pone.0079670)

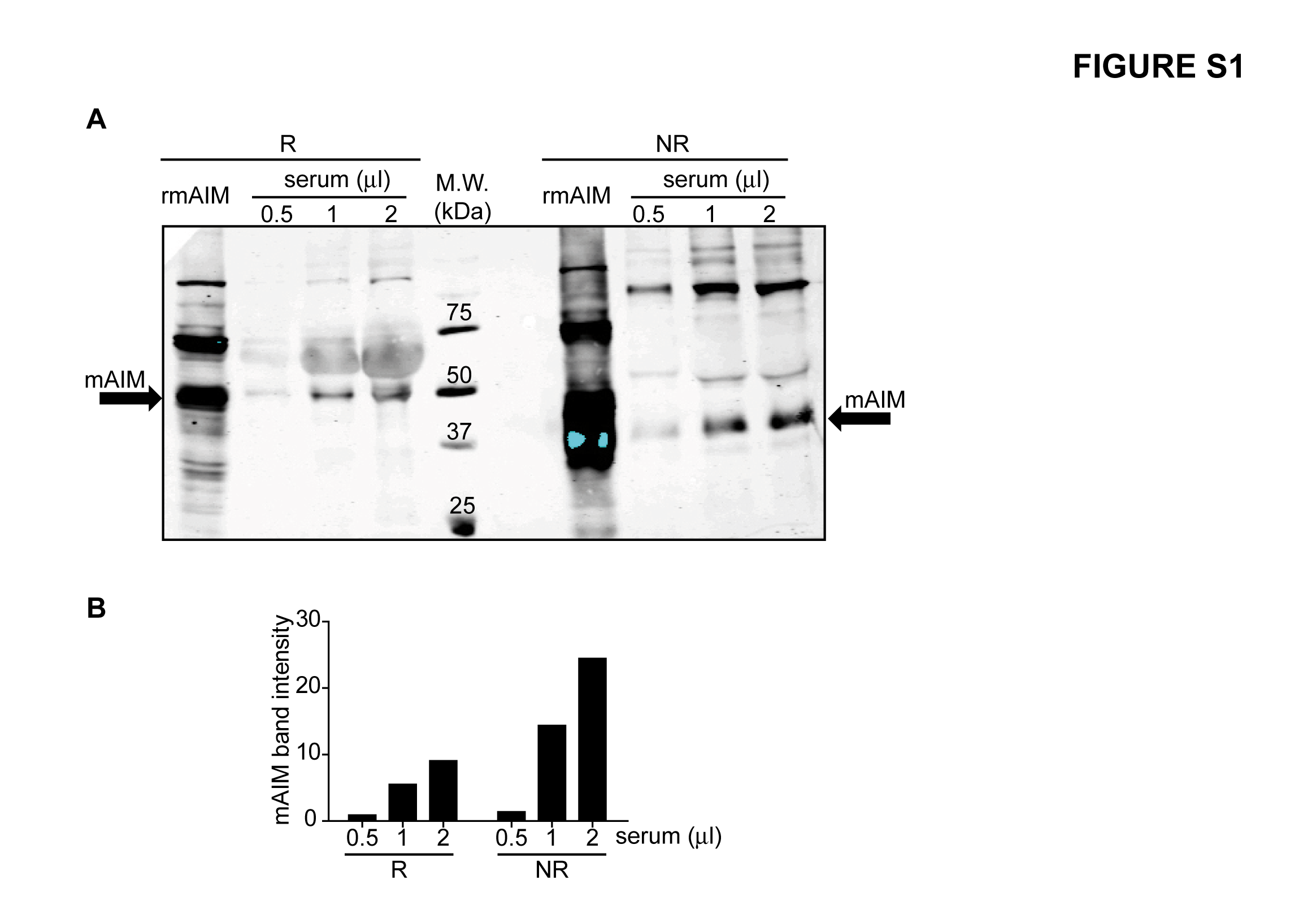

Supplement: Figure S1 — Optimization of mAIM detection in serum by western blot analysis. A) Representative image of mAIM detection analyzed by Western blot of serum samples. Either rmAIM or the indicated amounts of serum were resolved in 8% SDS polyacrilamide gels under R or NR conditions and the presence of mAIM was detected with an specific antibody. B) Graph depicting the results of the densitometric analysis performed using the Odyssey V.3 software (LI-COR). (TIF) [file pone.0079670.s001.tif]
