# Supplementary material for: The Scavenger Protein Apoptosis Inhibitor of Macrophages (AIM) Potentiates the Antimicrobial Response against Mycobacterium tuberculosis by Enhancing Autophagy
Source: PLoS One. 2013 Nov 4;8(11):e79670. doi: 10.1371/journal.pone.0079670 (PMC3817138; doi:10.1371/journal.pone.0079670)
Supplement: Protocol S1 — (DOCX) [file pone.0079670.s002.docx]

**PONE-D-13-22744**

**Protocol S1**

**Cloning and expression of recombinant mouse AIM.**

To obtain mAIM for its use as a positive control in the western blot experiments of infected mouse serum a recombinant form of the protein was expressed in the laboratory as follows. The cDNA of mAIM was obtained by reverse transcription of C57BL/6 mouse spleen mRNA (Clontech, Mountain View, CA, USA) and subsequent polymerase chain reaction (PCR) amplification with the following primers 5’ GCCCGGCTAGCGGAGTCTCCAACCAAAGTG 3’ (forward), 5’ : CGCGCGGATCCTCACACATCAAAGTCTG 3’ (reverse). The amplified PCR product was cloned into the pGEM-T vector (Promega, Madison, WI, USA), further NheI/BamHI-restricted and cloned into appropriately digested pCEP-4 vector (Invitrogen). Subsequently, the human embryonic kidney 293 (HEK293) cell line was transiently transfected with the pCEP-mAIM construct or empty pCEP-4 vector using the transfectin lipid reagent (Bio-Rad), following the manufacturer's instructions. Intracellular expression of mAIM in transfected cell lysates was assessed by western blot as detailed in the materials and methods section of the manuscript.

**Optimization of mAIM detection in serum by western blot analysis**

To optimize mAIM detection in whole mouse serum by western blot, different amounts of C57BL/6 mouse serum were analyzed under non-reducing (NR) and reducing (R, containing 25mM DithioThreitol) conditions, using the recombinant form of the protein (rmAIM) as a reference control. Samples were loaded into 8% SDS gels and immunodetection was performed as detailed in the materials and methods section of the manuscript. As observed in **Figure S1A**, western blot analysis of mouse serum showed that rmAIM used as positive control was detected at a molecular weight (MW) of 50kDa under R conditions. A similar reactivity at 50kDa was detected in mouse serum, suggesting that the antibody was specifically recognizing mAIM. Moreover, little cross-reactivity was observed around this MW. However, the mAIM band was too close to that of the immunoglobulin heavy chains as well as albumin, which did not facilitate quantification under these settings. The mAIM protein was best detected under NR conditions, in which both recombinant and serum forms presented a MW of 37 kDa. The differences in MW between R and NR conditions can be explained by the elevated number of cysteine residues of the SRCR domains of mAIM [56]. In these experiments, we also confirmed that the signal was dose-response dependent, given that loading different amounts of serum yielded increasing signal responses (**Figure S1B**).
